# Rectum Terminating in a Cul-de-sac

**Published:** 1849-05

**Authors:** 


*Rectum terminating in a Cul-de-sac.*—Mr. Wickliffe gave the following history of the case:—A female child, large, well nourished, and apparently well formed, born Aug. 23d, 1848, 10 A. M. Within three hours after birth the infant took the breast voraciously, when she fell asleep, and slept many hours. When she awoke she refused the nipple, and never sucked again.

On the following day the nurse reported that the child had often made water, but had had no motion. A dose of castor-oil was then given; this induced vomiting; oil, mucus, and a yellow sticky matter, were thrown up. The child now strained violently to stool. On carefully examining the anus, which was unusually open, and of triangular shape, it was found of a red aspect, somewhat like a sprout of fungoid growth. A cylinder of soap could be introduced about half a barley-corn in length, a blunt probe not farther, and the little finger passed as far. The abdomen had become tense. Thirty-five hours after birth a director was introduced as far as possible, and with a phymosis-knife an incision was made in the direction of the usual track of the rectum, the point and concave of the bistoury being directed upwards and backwards, to avoid, if possible, the vagina. The instrument was seemingly plunged into an elastic swelling; a few drops of blood only followed. Into the opening thus made, the canula of a trocar was introduced; this, at about an inch and a half, pressed against the elastic swelling before felt.—Through the canula a trocar was thrust; this was not, however, followed by meconium, but by a few drops of blood, and a drop only appeared at the orifice of the vagina, which was unusually large. A poultice was applied, and the child passed a quiet night, though she vomited occasionally. On the third day the child seemed little altered in appearance. Seventeen hours after the operation hæmorrhage came on from the vagina, and occurred at intervals for many hours. At ten o'clock P. M., the infant was tranquil, and died before midnight.

Examination twenty-three hours after death.—External appearances: A fine well nourished child; abdomen much distended; funis flattened, dried and transparent; a small clot of blood in the orifice of the vagina; the anus apparently patent. Opening the cavity of the abdomen, the first incision disclosed much fat and very distended intestine, the colon especially; there was no air in the cavity of the abdomen. The symphisis pubis was divided, and the bladder, uterus, and vagina removed, the posterior wall of the vagina being much infiltrated with blood, having been necessarily wounded in the operation. The rectum was entirely wanting. The colon terminated in the median line of the body in a cul-de-sac; it was much distended with meconium, which was slightly thinner, and not quite so dark-colored as usual; the appendix vermiformis caeci was unusually long. The uterus bi-angular rather than pyriform.—*Prov. Med. and Surg. Jour.*

